# Phenolic Compounds of the Medicinal Plants in an Anthropogenically Transformed Environment

**DOI:** 10.3390/molecules28176322

**Published:** 2023-08-29

**Authors:** Natalya Vinogradova, Elena Vinogradova, Victor Chaplygin, Saglara Mandzhieva, Pradeep Kumar, Vishnu D. Rajput, Tatiana Minkina, Chandra Shekhar Seth, Marina Burachevskaya, Dionise Lysenko, Rupesh Kumar Singh

**Affiliations:** 1 Department of Management, Economics of Pharmacy, Pharmacognosy and Pharmaceutical Technology, Federal State Budgetary Educational Institution of Higher Professional Education, M. Gorky Donetsk State Medical University, 283003 Donetsk, Russia; arina0vinogradova@yandex.com; 2 Laboratory of Dendrology of the Federal State Budgetary Scientific Institution “Donetsk Botanical Garden”, 283001 Donetsk, Russia; elena_vinogradova2021@mail.ru; 3 Academy of Biology and Biotechnology, Southern Federal University, 344006 Rostov-on-Don, Russia; otshelnic87.ru@mail.ru (V.C.); msaglara@mail.ru (S.M.); tminkina@mail.ru (T.M.); marina.0911@mail.ru (M.B.); 4 Department of Botany, Banaras Hindu University, Varanasi 221005, India; pradeep.kumar@bhu.ac.in; 5 Department of Botany, University of Delhi, New Delhi 110007, India; csseth52@gmail.com; 6 Faculty of Pharmacy, Saint Petersburg State Chemical and Pharmaceutical University, 197022 St. Petersburg, Russia; lysenkodionis@mail.ru; 7 Centre of Molecular and Environmental Biology, Department of Biology, University of Minho, 4704-553 Braga, Portugal; rupeshbio702@gmail.com

**Keywords:** heavy metals, anthropogenically transformed environment, phenolic compounds, flavonoids, anthocyanins, tannins and phenolic acids

## Abstract

In this article, the impact of an anthropogenically transformed environment on the content of pharmaceutically valuable biologically active compounds in medicinal plants is analyzed. The studied biologically active substances included phenolic compounds (flavonoids, anthocyanins, tannins, and phenolic acids). The number of transmissible forms of heavy metals (HMs), including cadmium, lead, and mercury, were discharged from factories that are present in the soil. Plants uptake these toxic metals from the soil. HM causes changes in the activity of the several enzymes such as phenylalanine ammonia lyase (PAL), chalcone synthase (CHS), chalcone isomerase (CHI) and other enzymes. These enzymes play an important role in biosynthesis of phenolic compounds in medicinal plants. It has been demonstrated that plant materials possess high antioxidant potential due to their high phenolic content. As a result, the present review discusses a thorough investigation of anthropogenically transformed environment effects on the quantity of pharmaceutically valuable phenolic compounds in medicinal plants.

## 1. Introduction

Despite all the advances in the production of synthetic drugs, the interest in herbal medicines has increased in the last few decades. In the modern pharmaceutical market, every third drug is of plant origin [1]. However, the traditional areas for harvesting medicinal plants are constantly decreasing as more and more lands are experiencing a technogenic load. This necessitates studying the possibility of using medicinal plants growing in anthropogenically transformed environments, particularly in urban areas. Our previous review [2] is devoted to the analysis of the environmental safety of medicinal plants harvested under such conditions. The review shows that although it depends on the biological characteristics of plants, the conditions of a particular region, the type and intensity of pollution, for some plant species it is possible to obtain uncontaminated medicinal plant materials even when growing in a contaminated area. However, in order to conclude on the potential possibility of expanding the zones of medicinal plant harvesting, it is necessary, in addition to environmental safety, to assess their pharmaceutical values.

Plants are characterized by significant variability in chemical composition depending on growing conditions. Therefore, when growing medicinal plants in an anthropogenically transformed environment, it is necessary to carefully study the content of biologically active substances (BASs), which determine their therapeutic effects. Many of these metabolites play a significant role in the formation of the ecological stability of plants, and therefore their concentration can vary significantly under the conditions of the technogenic environment. First of all, this concerns phenolic compounds, whose important function is to protect plants from adverse factors, which causes their biosynthesis to be more sensitive to technogenic pollution [3,4,5,6].

From the aforesaid, the purpose of this study was to systematize and analyze the information on the content of pharmaceutically valuable phenolic compounds in medicinal plant materials harvested under the conditions of technogenic load.

## 2. Phenolic Compounds

Unlike other secondary metabolites, phenolic compounds are found in almost all plant cells. They hold functional significance not at the cell level, but at the level of the whole plant. The central enzyme of phenylpropanoid metabolism (phenylalanine ammonia-lyase) is inducible: due to the induction of the expression of coding genes, its activity increases sharply under the influence of stress factors [3]. The antioxidant effect of phenolic compounds is due to the presence of aromatic rings with hydroxyl groups, is widely known. Under controlled conditions, these metabolites exhibit a higher ability to inhibit free radical oxidation than known non-enzymatic antioxidants such as ascorbic acid and α-tocopherol [6,7]. The example of transgenic plants *Solanum tuberosum* L. showed that an increase in the antioxidant potential correlates with an increase in the content of phenolic compounds, such as anthocyanins, flavonoids, and phenolic acids [8]. The activity of the multicomponent antioxidant system largely determines the resistance of plants to technogenic stress [5]; however, the polyfunctionality of phenolic compounds does not allow us to reduce their participation in the formation of plant tolerance only to their antioxidant activity. They are also cofactors for polyphenol oxidase and monooxygenases, and therefore take part in the functioning of the plant cell microsomal system responsible for the detoxification of xenobiotics [5].

The scientific literature quite often describes model experiments where plants are treated with salts of heavy metals (HMs) under controlled conditions [9]. *Aegiceras corniculatum* L. plants were treated with copper, zinc, and cadmium chlorides led to increased accumulation of phenolic compounds [9]. The example of *Dunaliella salina* (Dunal) Teodoresco shows an increase in the concentration of phenolic compounds, flavonoids and carotenoids caused by cadmium stress [10]. However, to study the possibility of using plants for medicinal purposes, it is necessary to conduct studies in natural conditions. In natural conditions, plants are exposed to the complex effects of various toxicants with the response to a certain extent depending on the soil and climatic conditions of a particular region. Leaves of *Vaccinium myrtillus* L. accumulated phenolic compounds when growing near a zinc–lead smelter [11]. An increase in the concentration of phenolic compounds in the needles of *Pinus halepensis* Mil was found under high concentration of sulfur dioxide in the air, while a negative correlation was found between the content of nitrogen oxides in the air and the total level of phenolic compounds in the needles, which apparently, is explained by the influence of these toxicants on the activity of nitrate reductase [12]. When grown in soil contaminated with copper and zinc sulfates, the content of phenols and flavonoids in *Avena sativa* L. seedlings decreased [13].

The study of the above-ground parts (for herbaceous plants) and leaves (for trees and shrubs) of 22 plant species in the urban ecosystem of Kaliningrad showed that herbaceous plants are the most sensitive to lead pollution in the soil. In most of the studied grass species, with an increase in the level of lead in the soil, an average decrease of 1.4–3.1 times was observed in the content of phenolic compounds. In shrubs, a decrease in the concentration of phenolic compounds under the influence of a pollutant was observed in the leaves of *Viburnum opulus* ‘Roseum., *Syringa vulgaris* L., *Ribes alpinum* L., *Philadelphus coronarius* L., and *Symphoricarpos rivularis* Suksdorf. In the zone of maximum pollution, it decreased by 1.3–1.9 times compared with the background. In the leaves of *Ligustrum vulgare* L., *Hippophae rhamnoides* L., and *Spirae vanhouttei* (Briot.) Zab., an increase in the content of lead in the soil stimulated the accumulation of phenols by an average of 1.3 times, and in the leaves of *Berberis vulgaris* L., *Sambucus nigra* L., and *Rosa rugosa* Thunb. at different sites, it did not have significant differences compared with the control. In the leaves of woody plants, the accumulation of polyphenols was multidirectional: in *Tilia cordata* Mill., and *Acer platanoides* L., a decrease in the content of phenolic compounds by 1.8–2.4 times was observed; in the leaves of *Betula pendula* Roth and *Populus nigra* L., their level increased by 1.7–2.8 times [5].

However, the study of the total content of phenolic compounds does not allow one to understand the complex mechanisms of regulation of the biosynthesis of individual groups of these substances by external conditions, and therefore there is no possibility of predicting the phytochemical composition of plants under specific conditions, especially introducing a medicinal species to a new habitat in order to obtain pharmaceutically valuable raw materials. It is important to note that numerous groups of phenolic compounds are united by a common biosynthetic pathway, and therefore their content changes in an interconnected manner. Changes in the metabolism of phenols are the most important mechanism for plant adaptation to technogenic load [4]. In plants, the total content of phenolic compounds may not change under the influence of toxicants, but the concentration of individual metabolites of this group may change [9,14]. Due to an increase in the total content of phenolic compounds, a decrease in the level of the phenolic metabolites that determine the therapeutic effects of the plant are possible. It also depends on the vital state of the plant, since the substances of secondary metabolism do not have their own synthesis pathways, and their biosynthesis occurs in the branches of the metabolic pathways of primary metabolites (e.g., proteins, carbohydrates, and lipids), where a wide range of enzymes function. From the aforesaid, we further considered the impact of technogenic load on the content of the most important phenolic metabolites in pharmacy flavonoids, anthocyanins, tannins, and phenol carboxylic acids.

### 2.1. Flavonoids

Flavonoids play a significant role in protecting plants against various kinds of stresses of a biogenic and abiogenic nature [3,12,15]. Appropriate literature analysis shows that the impact of technogenic pollution can affect the accumulation of these substances in plants in different ways.

**Figure 1:**
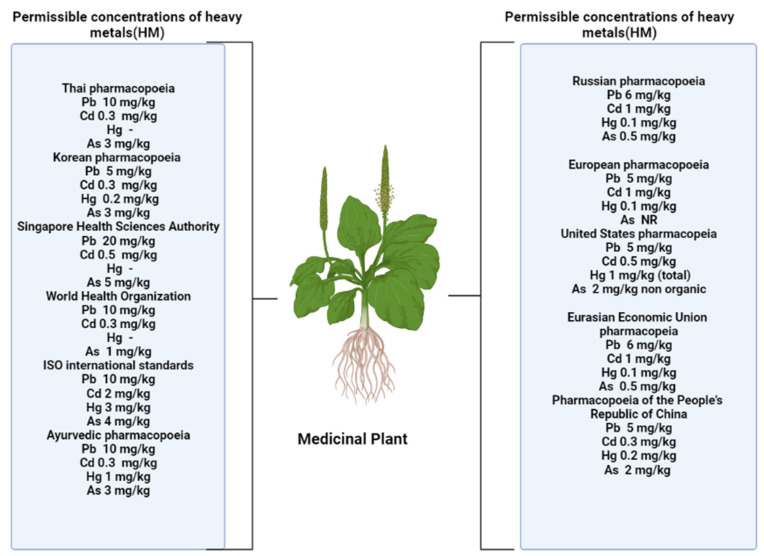
Permissible levels of heavy metals in accordance with standards established in various nations [2].

A number of authors have put forward the assumption that flavonoids contribute to the formation of plant tolerance to adverse environmental conditions. An increase in the content of these metabolites is one of the nonspecific reactions to stress [11,12,16,17]. Several pharmacopeia authorities fixed the HM concentration in different herbal formulation are shown in Figure 1.

Literature describes the possibility of using HM to activate the phenylpropanoid pathway. Induction of the synthesis of flavonoids was noted in the tissues of *Lemna gibba* L. plants under the influence of Cu^2+^, in the calluses of *Linum isitatissimum* L. and *Camellia sinensis* (L.) Kuntse treated with cadmium preparations, in *Phaseolus coccineus* L. treated with Cd^2+^ and Cu^2+^ [16,18]. In the study by Santiago et al. copper sulfate was used to induce the synthesis of phenolic compounds in *Phyllanthus tenellus* Roxb. [3]. The example of *Spinacia oleracea* L., showed that mercury salts treatment leads to an increase in the content of flavonoids in leaves due to a decrease in the total amount of phenolic compounds, which is explained by the effect of this toxicant on the phenylpropanoid pathway [19]. In the needles of *Larix sibirica* Ledeb, an increase in the total content of phenolic compounds (by 50–55%), flavonoids (by 1.5–1.8 times), catechins (by 1.9–2.5 times), and proanthocyanidins (by 45%) was noted compared with the background level. The described effects were observed at weak, moderate, and strong pollution levels due to large aluminum plant emissions, while at a critical level of pollution, the content of these metabolites decreased [20]. In the study of plants growing along the highways of the large city of Donbass, an increase in the concentration of flavonoids was revealed in the flowers of *Sambucus nigra* L., the fruit of *Rosa lupulina* Dubovik, as well as in the fruit, leaves, and flowers of *Crataegus fallacina* Klokov due to a decrease in the content of other phenolic compounds in the fruits of this species *Crataegus* [21].

There are often studies that describe directly opposite phenomena. The revealed decrease in the level of flavonoids under the influence of technogenic load in *Tussilago farfara* L., *Trifolium rubens* L., and *Vicia cracca* L. is explained by the participation of these metabolites in HM chelation, their antioxidant function, oxidation to quinones under the action of free radicals, and suppression of biosynthesis by toxicants due to damage to the structure of enzymes (synthetase and reductase) by reactive oxygen species (ROS) or HM [13]. There is a probable relationship between the lower photosynthetic capacity of *Tibouchina pulchra* Cogn. in the conditions of environmental pollution from emissions of fertilizer producers, metallurgical plants and the chemical industry, changes in carbohydrate metabolism, and a decrease in the concentrations of phenolic compounds and tannins [22]. At the same time, when exposed to industrial air pollution in a large city in *Latin America* (emissions from the chemical and steel industries, ferrous metallurgy, the production of fertilizers and ceramics) with high concentrations of fluorine, particulate matter, and sulfur dioxide, no significant differences were found in the total amount of flavonoids in the leaves of *Psidium guajava* L. compared with plants growing in an uncontaminated area [14].

In the leaves and flowers of *Tanacetum vulgare* L., *Chamaenerion angustifolium* (L.) Holub., leaves of *Linaria autiloba* Fisch. ex. Reichenb., *Artemisia mongolica* (Bess.) Fisch. ex. Nakai, *Artemisia jacutica* Drob., *Inula britannica* L., *Taraxacum ceratophorum* (Ledeb.) DC., *Achillea millefolium* L., a significant decrease in the concentration of flavonoids was revealed in conditions of cement dust pollution (in the vicinity of a cement plant) [15]. In the study of plants growing along the highways of the capital of Donbass, a decrease in the concentration of flavonoids was found in the leaves of *Cotinus coggygria* Scop, the fruit of *Rosa corymbifera* Borkh., *Sorbus aucuparia* L. and *Sorbus intermedia* (Ehrh.) Pers [23].

The study of the characteristics of flavonoid metabolism in wild plants forced to adapt to an environment polluted by industrial emissions is also important for studying the formation of tolerance in specific species to technogenic stress and can be used to diagnose the state of the environment. In the study of plants *Trifolium pratense* L., *Artemisia absinthium* L., *Taraxacum officinale* L., and *Achillea millefolium* L. under the conditions of their long-term adaptation to technogenic pollution (at the Ufa oil refinery and its surroundings), a lower content of flavonoids in the aerial part was found compared with plants from an ecologically clean zone. The results obtained correlated with the level of soil contamination. At the same time, another pattern was revealed in the roots of the studied plants–the content of flavonoids was higher for plants from the contaminated area [24]. It is shown that under conditions of varying degrees of pollution in the plant, both an increase and a decrease in the content of flavonoids can be observed (for example, *Matricaria chamomilla* and *Poa pratensis*), as the authors suggest, this indicates a wide range of resilience of these species [13].

It is important to note that the method of quantification of flavonoids (different authors often use different methods) can affect the results obtained. It should also be taken into account that under conditions of technogenic load, hydrolysis of glycosidic forms of flavonoids can occur, and can lead to an increase in their recorded content [13]. The place of harvesting of the control plants with which the comparison is made is also important. As such, various researchers use a botanical garden, a park, the countryside, as well as pharmacy plant materials. Often, an article does not provide a detailed description of the environmental conditions for the growth of control plants, although the content of biologically active substances is affected not only by the technogenic load, but also by the composition of the soil, humidity, temperature, amount of solar radiation, etc. This makes it difficult to interpret the results.

#### Mechanism of Heavy Metals in Enhancement of Phenolic Compound

A significant worldwide concern is the release of HM into the ecosystem as a result of various anthropogenic activities. The growth of medicinal plants in soil enriched with HM can ultimately impact the biosynthesis of phytocompounds [25,26]. These HM is be responsible for a higher level of ROS generation. Due to increasing ROS generation, it may cause oxidative stress, deterioration of biomolecules, and breakdown of the lipid membrane.

**Figure 2:**
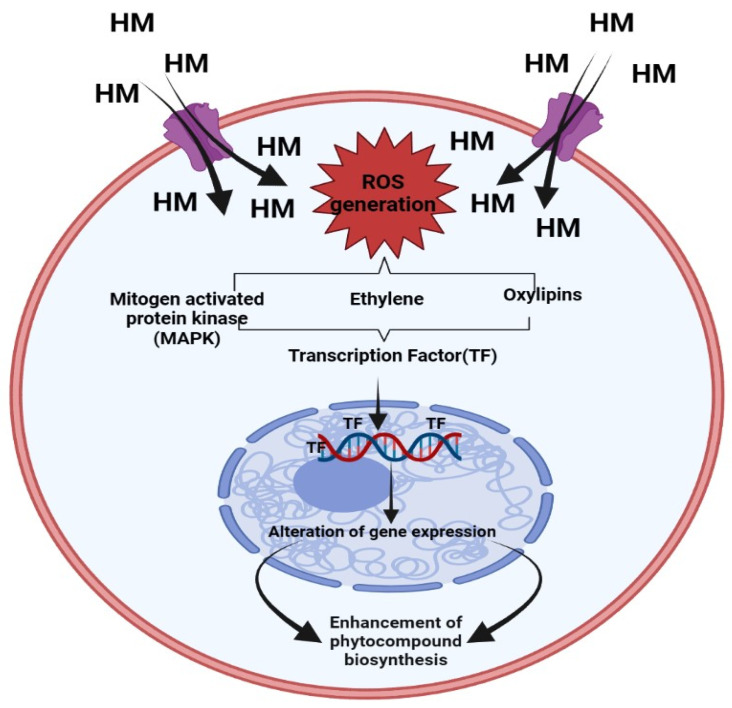
Mechanism of heavy metals in enhancement of Phenolic compound [26].

On the other hand, ROS also function as messenger molecules, which may trigger other downstream signaling channels that regulate phytocompound synthesis inside medicinal plants. It has been discovered that higher levels of ROS brought on by HM stress cause mitogen-activated protein kinases (MAPKs) to become active. Directly phosphorylating the higher expression of transcription factors like CrMAPK3 and ORAC3, in turn causes an increase in quantity of various phyto-compounds of *Catharanthus roseus* under Ag^+^ treatment via increasing expression of GS, STR, and CrPRX as well as DAT genes [27]. It is also widely known that HM stimulates the expression of other signaling molecules such as ethylene, which participates in the regulation of pathways that lead to the synthesis of tropane alkaloids (hyoscyamine and scopolamine) in *Brugmansia candida* hairy root cultures subjected to Ag^+^ [28]. A diagrammatic representation of the signaling route involved in the buildup of phytochemicals brought on by heavy metals is represented in Figure 2.

HM stress leads to the production of toxic ROS in plants, which in turn results in poisoning as well as diminished plant development [29,30,31]. Several studies reveal that HM is responsible for the biosynthesis of polyphenols, which act as shields for plants against oxidative damage [32,33,34]. The finding reveals that the amount of polyphenols in plants has been shown to be significantly boosted by metal excess [34,35], which aligns well with the finding that polyphenols may accelerate the metallic chelation mechanism and assist in reducing the amount of damaging free radicals in plant cells [36,37]. High exposure to HM causes the accumulation of bioactive compounds like flavonoids that help the defense systems of plants [38,39,40]. Under metal stress the level of transcription of the genes of such enzymes as phenylalanine ammonia lyase (PAL), chalcone synthase (CHS), chalcone isomerase (CHI), cinnamate 4-hydroxylase (C4H), 4-coumarate: CoA ligase (4CL), flavanone3-hydroxylase (F3H), flavonoid 30-hydroxylase (F30H), flavonoid 305 0-hydroxylase (F305 0H), flavonol synthase (FLS), flavone synthase (FNS), UDP flavonoid glycosyl-transferase (UFGT), isoflavone synthase (IFS), isoflavone reductase (IFR), dihydroflavonol 4-reductase (DFR), anthocyanidin synthase (ANS) [40].

**Figure 3:**
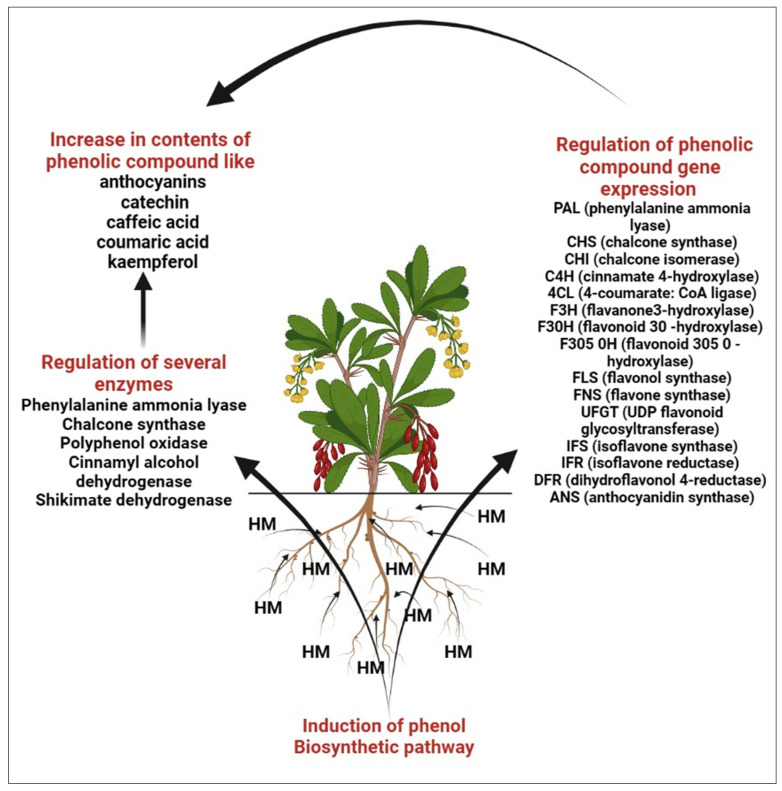
Role of heavy metal in enhancement of phenolic compound.

**Figure 4:**
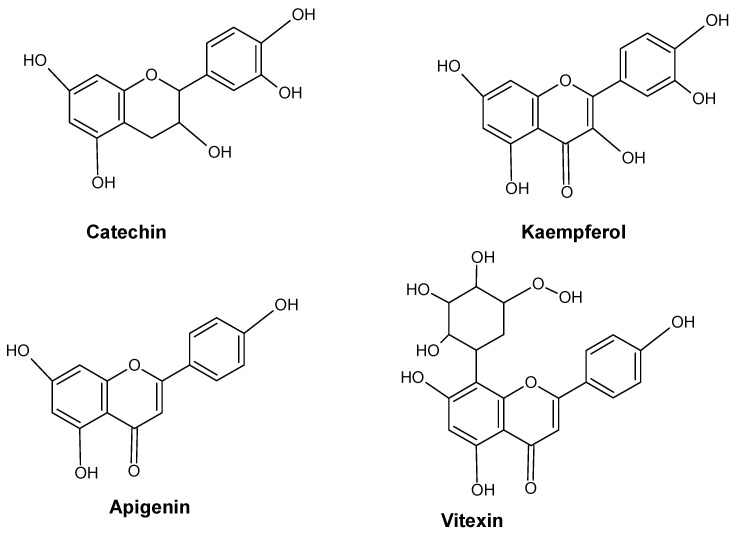
Major flavonoids in plants.

In the phenolic/ascorbate-peroxidase cycle, flavonoids are known for their ability to scavenge H_2_O_2_ and are thought to be essential [41,42,43]. Several significant enzymes, including shikimate dehydrogenase (SKDH) and glucose-6-phosphate dehydrogenase (G6PDH), facilitate the biochemical process needed to produce key substrates of phenylpropanoid pathways [44]. By increasing the activity of important biosynthesis enzymes such as PAL, SKDH, G6PDH, and CADH, heavy metals drive the phenylpropanoid biosynthetic pathway in plants [44,45]. The effects of metal stress on plants’ phenolic content are summarized in Figure 3. Several major flavonoids like catechin, kaempferol, apigenin and vitexin are shown in Figure 4.

### 2.2. Anthocyanins

Anthocyanins deserve special attention as a group of flavonoids due to their high physiological activity. It is suggested that, in contrast to pigmentation in flowers and fruit, the accumulation of these metabolites in leaves is a response to oxidative stress [46]. In addition to the direct neutralization of free radicals (their antiradical activity exceeds that of other classes of flavonoids, ascorbate, and α-tocopherol), anthocyanins are capable of chelating metal ions [47]. Anthocyanins located in vacuoles are able to neutralize peroxides, which is especially important due to the fact that the absence of ascorbate peroxidase in cell vacuoles does not allow ascorbic acid to participate in the detoxification of ROS [48]. Not vacuolar, but cytosolic anthocyanins likely make a more significant contribution to the antioxidant system; they are located closer to the sources of superoxide radical synthesis. The degree of contribution of anthocyanins to the antioxidant system of the plant differs among other low molecular weight antioxidants in different species. In some plants, they are the main antioxidants, while in others, a high level of anthocyanin biosynthesis is not necessary for protection against oxidative stress [49].

The biosynthesis of anthocyanins is a nonspecific response to stressful growing conditions (low temperatures, drought, HM pollution, oil pollution, and ionizing radiation), and can be explained by the expression of genes associated with their synthesis [5,50,51]. The content of anthocyanins is proposed to be used to assess the physiological state of plants [5].

An analysis of literary sources shows that most researchers note the accumulation of anthocyanins in the leaves of plants growing under conditions of technogenic pollution. Under conditions of oil pollution, an increase in the content of anthocyanins was detected in the leaves of *Leymus arenarius* L. Hochst, *Ammophila arenaria* L. Link, *Lathyrus maritimus* Bigel., *Petasites spurious* (Retz) Reishb., *Salix daphnoides* Will., *Salix caprea* L., and *Salix aurita* L. [5,52]. Under conditions of pollution with railway pollutants (petroleum and transmission oils), an increase in the concentration of anthocyanins was found in the leaves of *Geum urbanum* L., *Anthriscus sylvestris* L., *Glechoma hederacea* L., *Taraxacum officinalis* L., *Dactylis glomerata* L., and *Achillea millefolium* L. (by an average of 5.2 times). Cultivation of *Cassia grandis* L. plants on a mining waste substrate for 4 months led to an increase in the concentration of anthocyanins in leaves and roots (due to a decrease in the content of chlorophyll a, b and carotenoids), where the control plants were grown on a commercial substrate [51]. In urban conditions, an increase in the content of anthocyanins in the leaves of *Psidium guajava* ‘Paluma’ was found [53].

In the conditions of the urbanized environment of Donbass, anthocyanin accumulation was observed in the fruitof *Rosa corymbifera*, *Sorbus aucuparia*, and *Sorbus intermedia*, flowers and leaves of *Crataegus fallacina*, while in the fruit of *Rosa lupulina* and flowers of Sambucus nigra their concentration decreased [21,23]. With the intensification of urban air pollution by automobile pollutants, the content of anthocyanins increased in the needles of *Picea abies* (L.) Karst., leaves of *Tilia cordata* Mill., *Taraxacum officinale* Webb., Plantago major L., *Sorbus aucuparia* L., and *Acer platanoides* L. An increase in the concentration of cadmium in soil stimulated the accumulation of anthocyanins in the leaves of *Lathyrus maritimus* Bigel., *Secale cereale* L., *Lolium perenne* L., *Festuca rubra* L., and *Poa pratensis* L. was due to a decrease in the total content of phenolic water-soluble antioxidants [5,52].

**Figure 5:**
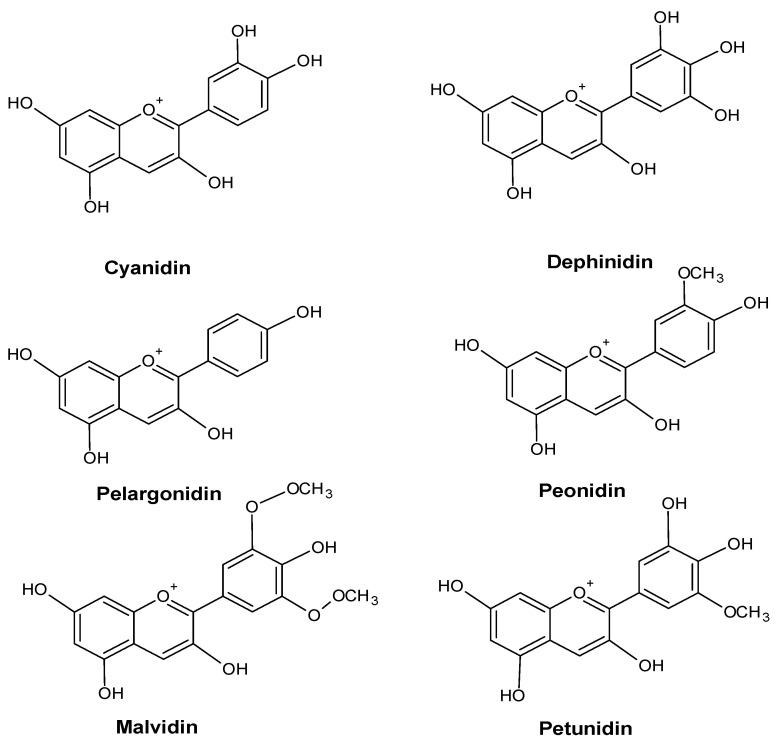
Major anthocyanin in plants.

An increase in the anthocyanin content of a plant’s leaves is frequently a sign of environmental stress. For example, Vasconcelos et al. showed the accumulation of anthocyanins in the leaves of *Zea mays* L. plants growing under conditions of phosphorus deficiency [50]. Some anthocyanins are shown in Figure 5.

### 2.3. Tannins

Many studies have noted a decrease in the content of tannins with an increase in the intensity of technogenic pollution, which is explained by the suppression of the processes of synthesis and the ability of metals to reduce the content of tannins by precipitation. In particular, a decrease in the level of tannins was found in the leaves of *Acer negundo* L., *Acer platanoides* L., and *Tilia cordata*, growing on plantations in sanitary protection zones of industrial enterprises and along the highways, in the leaves of *Prunus padus* L., collected next to a metallurgical enterprise and by a combined heat and power plant, in the leaves of *Plantago major* L., growing by a highway, in the leaves of *Betula pubescens* spp., growing near a steel plant, and in the aerial part of *Achillea nobilis* L. and *Melampyrum pratense* L. in the area of industrial installations of a gas processing plant [53,54,55]. The chelating ability of tannins is proposed to be used to remove metal ions from wastewater [56].

Under urban conditions, an increase in the content of tannins was found in the leaves of *Psidium guajava* ‘Paluma’ [53]. In the leaves of *Acer platanoides* L. plants growing on the plantations in the sanitary protection zones of the casting and blacksmith plants and along the highways, an increase in the activity of polyphenol oxidase was revealed compared with the control (plantations of the forestry), while the content of condensed tannins decreases, which indicates the active participation of tannins in adaptive reactions of plants associated with the mechanisms of neutralization of pollutant effects [54].

Katoh et al. showed that the inhibition of the synthesis of condensed tannins in the leaves of *Cryptomeria japonica* D. Don., caused by air pollution in the vicinity of a steam power plant, is due to disturbances in the formation of aromatic rings from 3-dehydroshikimic acid, i.e., the conversion of shikimic acid to phenylalanine and tyrosine [57]. The content of tannins in the leaves is negatively correlated with the levels of soluble sulfate. Under the influence of toxicants, a decrease in the photosynthetic activity of *C. japonica* is observed. The authors suggest that in this case the main part of free glucose is used for growth and primary metabolism, which can explain the decrease in the intensity of secondary metabolism, including synthesis of tannins [58].

Along with the above results, there are also other studies showing an increase in the biosynthesis of tannins in contaminated areas, which is explained by the protective reaction of plants to adverse environmental conditions. This is observed in the leaves of *Fragaria viridis* L. plants growing on the territory of the gas processing plant, and in *Plantago* samples taken near the highway [53,55,58]. Under the conditions of the urbanized environment of Donbass, a decrease in the concentration of tannins was found in the medicinal raw material of most of the studied species (*Rosa lupulina*, *Sorbus aucuparia*, *Sorbus intermedia*, *Sambucus nigra*, *Tilia cordata*, *Cotinus coggygria*), while in the fruit of *Rosa corymbifera*, leaves and flowers of *Crataegus fallacina* its content increased [21,23].

**Figure 6:**
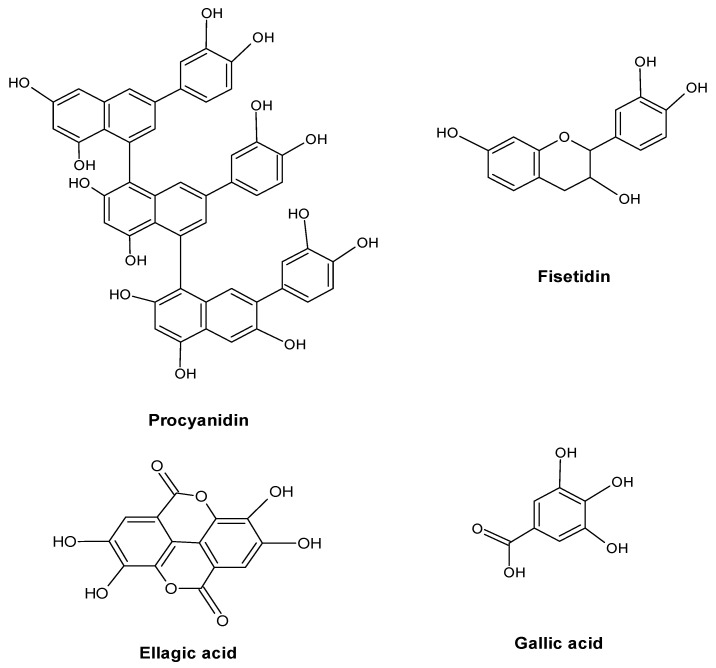
Major tannins in plants.

Thus, the dynamics of the content of tannins under conditions of pollution requires careful study for each individual type of medicinal plants in a particular region. The revealed discrepancy in the results observed by different authors can also be explained by the predominant group of tannins (hydrolysable or condensed) in the studied plant material and, accordingly, by different ways of their biosynthesis and properties, which determine the reaction of their metabolism to technogenic impact. A good example of this is the work that analyzed the concentration of tannins in the fruit of *Rosa* L., grass *Tussilago farfara* L. and *Plantago major* L., harvested near the highway [55]. A decrease in the concentration of hydrolysable tannins compared with the control (forest) was revealed, which is explained by their binding to HM, and an increase in the content of condensed tannins due to their protective function (Figure 6).

### 2.4. Phenolcarboxylic Acids

Free phenol carboxylic acids, along with flavonoid aglycones, are the most physiologically active forms of phenolic compounds [4]. Of these, hydroxycinnamic acids are especially significant in pharmacy, having a variety of pharmacological effects on the human body. In plants, they are localized in the cell wall and play an essential role in the regulation of its physico-chemical properties. In the case of mechanical damage or penetration of pathogens, hydroxycinnamic acids, and lignin, by binding to non-phenolic polymers of cell walls, they contribute to their strengthening, prevent the penetration of pathogens and uncontrolled loss of water. The ability of these metabolites to bind HM ions into stable complexes was found. Interestingly, the use of exogenous hydroxycinnamic acids to increase plant tolerance when growing under conditions of HM intoxication is a promising direction in soil reclamation. High antioxidant activity was noted for chlorogenic and ferulic acids [4,16,59].

Appropriate literature analysis shows that since various acids differ significantly in their functions, particularly in antioxidant activity and chelating ability, it is more informative to study not their total content, but the concentrations of individual acids. Under the conditions of the urbanized environment of Donbass, a decrease in the concentration of hydroxycinnamic acids was found in the fruit of *Crataegus fallacina*, *Rosa lupulina*, *Sorbus aucuparia*, and *Sorbus intermedia*, while in other types of medicinal raw materials harvested under the same conditions (leaves and flowers of *Crataegus fallacina*, leaves of *Cotinus coggygria*, flowers of *Sambucus nigra*), the concentration of these metabolites decreased [21].

In the leaves of *Matricaria chamomilla* L., under the influence of Ni treatment, the accumulation of chlorogenic acid (antioxidant) increases four times, while the content of protocatechuic acid, which has a high chelating ability, decreases [60]. A minimum amount of chlorogenic acid derivatives was found in the herb *Leonurus quinquelobatus* Gilib, collected in the zone of moderate pollution where the plants are the largest, while the samples from the control and severe pollution areas differed insignificantly for this indicator [16]. The example of *Hypericum perforatum* L. shows an increase in the level of some phenolic acids (for example, ferulic) in shoots and roots due to a decrease in the content of flavonoids. A possible explanation for this is the activation of the first stages of the synthesis of hydroxycinnamic acids, which is more preferable for plants, which is accompanied by a decrease in the activity of genes involved in the subsequent stages leading to the formation of flavonoids and anthocyanins. This is a way to save energy when it is enough to deal with stress [59].

In the study of Karpova et al. the leaves of *Spiraea media* Fr. Schmidt, *Spiraea chamaedryfolia* L., and *Spiraea hypericifolia* L. are analyzed when growing in areas of Novosibirsk with high and background levels of industrial pollution [4]. The content of the total phenolic compounds in the leaves of the studied species under pollution conditions is reduced in comparison with the plants growing in an ecologically clean zone. The decrease in the content of phenolic compounds in *S. media* occurred due to flavonol glycosides (mainly rutin), in *S. chamaedryfolia* and *S. hypericifolia*, due to hydroxycinnamic acids. In the leaves of *S. media* from an urban area, an increase in the concentration of p-coumaric and o-coumaric acids and a decrease in protocatechuic and p-hydroxybenzoic acids were revealed. In the leaves of *S. chamaedryfolia* from an urban planting, the concentration of the dominant cinnamic acid increased compared with the control (due to a decrease in the content of the other studied phenolcarboxylic acids and the total content of phenolic compounds). In the leaves of *S. hypericifolia* from an urban planting, the content of most components is reduced compared with the control samples, and only the content of protocatechuic acid is increased. The qualitative composition of phenolic compounds also changed, most significantly in *S. media*: the leaves of urban plants lack free quercetin and kaempferol and contain only half of the flavonol glycosides found in the leaves of plants from the control area. In the complex of phenolic compounds in leaves harvested under background conditions, flavonoid glycosides dominated, and in urban conditions, phenolcarboxylic acids dominated. The opposite pattern was observed in the leaves of *S. chamaedryfolia*. In the leaves of *S. hypericifolia*, both values did not change significantly. According to the authors, this indicates a greater adaptive potential of *S. chamaedryfolia* and *S. hypericifolia* due to the revealed tendency to inhibit the activity of metabolic processes [4].

Thus, the change in the level of phenolcarboxylic acids in plants as a result of exposure to technogenic pollution depends both on the predominant acid and on the overall metabolism of phenolic compounds and the level of physiological activity of the plant as a whole.

## 3. Nonmonotonic Dependences of the Content of Phenolic Compounds on the Technogenic Load

An analysis of the rich factual material accumulated in literature indicates that some plant species (probably more resistant) are characterized by a two-phase dependence of the content of phenolic compounds on the intensity of the technogenic load. As long as the pollution does not exceed the norm of the reaction of a particular type, an increase in the content of these metabolites is observed, and with a stronger technogenic pollution, a decrease is observed. It has recently been suggested that it is possible to achieve an increase in yield and the content of pharmacologically valuable substances in some types of plants when grown on soil contaminated with HM (subject to mandatory control of environmental cleanliness) [15,61,62]. This is very promising for plants whose medicinal raw materials are fruit, seeds, or flowers, i.e., parts characterized by the least accumulation of toxicants. The possibility of growing *Polygonatum sibiricum* plants in soil contaminated with cadmium was shown, which makes it possible to obtain tubers with a high content of active substances, polysaccharides [61]. Similarly, an increase in the quantity and quality of the essential oil of *Cymbopogon citratus* (D.C.) Stapf. when grown in soil supplemented with red mud and sewage, and the essential oil content of Melissa officinalis were highest when grown in the vicinity of a lead-zinc smelter [63]. However, these positive phenomena were observed only up to a certain concentration of toxicants, and then the inhibition of plant metabolism began. It has also been shown that the positive role of xenobiotics for the induction of the synthesis of secondary metabolites weakens with prolonged stress [61,64].

The reaction described above (the stimulating effect of low doses of the toxicant) is hormetic. The nonmonotonic dependencies of the BAS content on the intensity of the technogenic load also include a paradoxical dependence (an increase in the dose of a toxicant leads to a decrease in its damaging effect) [65]. Presumably, such dependencies are typical for metabolites directly involved in plant adaptation to environmental conditions [66].

The study [21] revealed a hormetic dependence of the content of anthocyanins and procyanidins in the leaves and flowers of *Crataegus fallacina*: under conditions of a moderate technogenic load, their concentration increases, and under a strong one, it no longer has a significant difference with the control. Such a hormetic dependence is probably due to the switching of plant resources to the synthesis of phenolic metabolites that are more effective under these conditions. A similar pattern was noted in the leaves of *Betula pendula* Roth.: lead in low doses caused an increase in the content of carotenoids, and high doses reduced this indicator [66].

Nonmonotonic dependencies can be observed not only in leaves, but also in other types of raw materials. The dependence of the concentration of anthocyanins and carotenoids on the degree of technogenic load in the fruit of *Crataegus fallacina* was ambiguous: under conditions of moderate pollution, their content was significantly lower than the control, and under conditions of severe pollution it significantly exceeded it. This is probably due to the ability of *Crataegus fallacina* plants to function in several adaptive modes depending on the strength of the stress factor. The same relationship was observed in the fruit of *Sorbus intermedia* for the content of carotenoids, and ascorbic and free organic acids, which may indicate the activation of additional protective mechanisms during the accumulation of HM by the plant, when the enhanced biosynthesis of anthocyanins is no longer enough to normalize vital activity [21]. A similar dependence is described by Erofeeva E.A. for the content of photosynthetic pigments in *Betula pendula* Roth, *Taraxacum officinale* Wigg., *Triticum aestivum* L. under polluted conditions [66,67]. A change in the level of certain phenolic metabolites is often recommended for bioindication of the state of the environment, since the dynamics of biologically active substances make it possible to detect the first stages of pollution, before any visual signs of damage appear [5,11,12,21,53,55,58]. However, with such recommendations, the authors often do not take into account the possibility of nonmonotonic dependencies, which can lead to an incorrect assessment of the quality of the environment. Such dependencies may go unnoticed due to the small number of zones selected for study.

## 4. Safety of Medicinal Plants, Growing under Technogenic Pressure

Of course, it is necessary to remember that medicinal plant materials harvested under conditions of technogenic load can be a source of various toxicants entering the human body and damage human health. According to the regulatory documentation of most countries when assessing the environmental safety of medicinal plant materials, the content of such heavy metals as lead, cadmium, and mercury is normalized. At the same time, the standards established by different countries differ somewhat. According to the State Pharmacopoeia of the Russian Federation the concentration of lead in medicinal plant materials and herbal medicinal products should not exceed 6.0 mg/kg, 1.0 mg/kg of cadmium, and0.1 mg/kg of mercury [68]. The European Pharmacopoeia establishes the following standards: 5.0 mg/kg of lead, 1.0 mg/kg of cadmium, and 0.1 mg/kg of mercury [69]. According to the United States Pharmacopoeia the content of lead should not exceed 5.0 mg/kg, 0.5 mg/kg of cadmium, and 1.0 mg/kg of mercury, and the content of methylmercury is separately regulated (not exceed 0.2 mg/kg) [70].

There is large number of publications that analyzes the regional features of HMs’ accumulation in medicinal herbs. Many medicinal plants growing under conditions of technogenic load accumulate HM in amounts that are many times higher than the permissible concentrations. However, for certain plant species, safety has been shown in terms of the HMs’ content of their medicinal plant materials harvested in urban ecosystems [21,23,71,72,73]. For example, the concentration of cadmium, lead, and mercury in medicinal raw materials of *Sorbus aucuparia* L., *Sorbus intermedia* (Ehrh.) Pers, *Rosa corymbifera* Borkh., *Crataegus fallacina* Klokov, and *Sambucus nigra* L., growing in the urbanized environment of Donbass, did not exceed the exceed the permissible concentrations, even for plants of first-row planting along urban highways with heavy traffic, and the contents of active substances met the requirements of regulatory documentation; while the leaves of *Cotinus coggygria* Scop were harvested under the same conditions, lead was accumulated in concentrations nine times higher than the permissible [2,21,23].

It is very important which part of the toxicants passes from the plant material into various dosage forms. For example, *Hypericum perforatum* L. is a super-concentrator of cadmium; however, the maximum extraction into decoctions did not exceed 23%, and 5% of its content in the herb into tinctures, which was due to the formation of physiologically inactive complexes [74]. The literature data on the extraction of HM by various extractants are very contradictory [16,75,76,77]. When assessing environmental safety, the dosage and duration of particular drugs use are important. Thus, it is necessary to approach with caution the results of studies showing an increase in the content of pharmacologically valuable phenolic compounds in medicinal plants growing in conditions of technogenic pollution. It is necessary to carry out a comprehensive check of medicinal plants growing in a specific territory, analyzing their environmental safety and medicinal value, while researchers often limit themselves to only one of these aspects.

## 5. Conclusions

To conclude on the suitability of a particular anthropogenically transformed area for growing medicinal plants, it is necessary not only to analyze their environmental safety, but also to evaluate pharmaceutical values. Under conditions of technogenic load, the content of phenolic compounds in plants changes species-specific, which is largely determined by the degree of contribution of one or another group of phenolic metabolites to the antioxidant system of a particular species, along with other low molecular weight antioxidants. The induction of the synthesis of a specific group of phenolic compounds as an adaptive response of plants to pollution can be considered a characteristic feature of the species. The impact of technogenic pollution leads to a change not only in the quantitative, but also in the qualitative composition of phenolic compounds. There is a potential possibility of using changes in the content of phenolic compounds for bioindication of the state of the environment and analysis of plant tolerance to stress conditions; however, it is necessary to take into account the possibility of nonmonotonic dependencies. It is necessary to study the content of pharmaceutically valuable compounds in medicinal plants across a wide gradient of technogenic load. For a large number of plant species, not only leaves, but other types of raw materials (fruits, flowers, and underground organs) are used to manufacture medicines, while most of biological research is devoted to study the changes in the chemical composition of leaves under conditions of technogenic load. This is logical since leaves are most susceptible to pollution due to metabolic activity, and a high surface area of interaction with air. However, due to the unequal response of various plant organs to stress, such studies are not enough to conclude on the suitability of a particular area for growing medicinal plants for their use in pharmacal industries. This substantiates the relevance of conducting a comprehensive phytochemical study of medicinal plant species in a particular region, not limited to leaves.

## Data Availability

Not applicable.
